# Annotations

**Published:** 1906-05-05

**Authors:** 


### ANNOTATIONS.

### Compulsory Vaccination.

Those who last week urged in the House of Commons the removal of the very moderate pressure towards vaccination, which is all that is left to us in this hey-day of liberty, were as liberal with fallacies of fact and inference as gentlemen of that kidney have ever been. Much was made of dissensions among the members of our profession as to the utility of vaccination as a prophylactic, with the object of establishing the insecurity of its reputation. It is true that medical opinion is not unanimous; it is too much to expect that every one of some forty thousand medical men in this country and its dependencies should be open to the sweet voice of reason. The minority is so minute as to be negligible. The chief importance of the dissentients from the generally received opinion lies in the readiness of the anti-vaccinationist to claim for them an exaggerated weight and dignity, as in this case in point. It was also urged in the House that those who credit the prophylactic power of vaccination should be content to use it, without forcing the noxious thing upon unbelievers. The inference is that the contagion of small-pox is to be regarded as a matter of purely personal interest, each man having the right to behave towards it in the way which seems most expedient to him. Unfortunately experience has demonstrated the fallacy of this reasoning. It has shown that the outbreak of small-pox in any community is apt to become a very expensive amusement for ratepayers, both on account of the dislocation of business and the actual out-of-pocket expenses of the sanitary departments. It is a hard fate which calls upon the protected vaccinationist to pay for his wilful neighbour's cranks. If in any epidemic of the disease the charges of the unvaccinated victims were borne by the anti-vaccinationists, and those of the vaccinated sufferers by the vaccinationists, we should soon hear much less of conscientious objection.

### Anthropology at Oxford.

The University of Oxford has recently instituted a diploma in anthropology, which is to be open to all persons, whether members of the University or not, who shall pursue an approved course of study and pass certain prescribed examinations. The term of study is fixed at one year as a minimum; but even this is not necessarily to be continuous, so that the new diploma may be within reach of administrators, missionaries, and other residents among non-European peoples who may decide to spend their "long leave" amidst the anthropological opportunities existing at Oxford. In addition to their scientific interest, it is now well recognised that anthropological studies are of great practical importance to all concerned with the government and instruction of native peoples and civilisations, and thus it is fitting that their development should be cultivated by a University which sends so many of her sons as public servants to the various parts of the British Empire. A word of recognition is due to the liberal spirit by which encouragement is offered to others than members of the University. Upon these no demand is made, except, as stated above, and the guarantee of "a good general education," a condition which, it may be presumed, will be interpreted in no narrow or technical sense. Oxford, it must be remembered, has very special and peculiar attractions for students of anthropology. The magnificent collection of the late Lieutenant-General Pitt-Rivers forms a museum specially adapted to the comparative study of the arts of mankind, both utilitarian and æsthetic, and particularly from the point of view of their development and geographical detail. In the Ashmolean Museum are the materials for studying the prehistoric periods, more especially in Europe and the Mediterranean basin; the collection of prehistoric Egyptian objects is said to be unique outside Egypt itself. The Indian Institute contains a museum arranged so as to give a synopsis of Indian life and customs, and is thus capable of furnishing valuable aid in the historical study of Indian subjects. The proposal to recognise by a university diploma the systematic study of these valuable records, and to make them accessible to all whose work they may aid, is one which will excite the sympathy of all cultured and liberal-minded persons.

### The London Polyclinic.

The Medical Graduates' College and Polyclinic held its seventh annual meeting some few weeks ago, and the occasion was appropriately selected for a review of the career of the institution by those responsible for its organisation. The position broadly is contained in the remark that, as shown by the last two balance-sheets, the College is now able to pay its way, but it is burdened by the debt incurred in the earlier years of its existence for the purposes of organisation and equipment. Were this debt removed, and the interest which now has to be paid on it consequently cancelled, the College would be able, not only to continue, but to extend its usefulness free from all financial liability. The sum at stake is not a large one (some £8,000, we understand), and might surely be realised by an appeal addressed to the proper quarters. The claim, it will be observed, is not one to meet annual charges, which must necessarily be repeated, but is one to remove a burden from an institution now conducted on a basis of solvency. It is satisfactory to gather from the annual report that the various teaching schemes of the Polyclinic are enjoying an abundant success, and the work which is included in these is certainly of high professional and general value. That the several clinics and practical classes are open to the profession on the payment of very modest fees is an additional reason why an effort should be made to get free from debt, for what increases the efficiency of the profession is directly conducive to the public interest. It is to be hoped that the efforts of the Council will meet with a substantial endorsement, and that the Polyclinic will thus be able to enlarge the sphere of its operations.

